# Tetrapod species–area relationships across the Cretaceous–Paleogene mass extinction

**DOI:** 10.1073/pnas.2419052122

**Published:** 2025-03-25

**Authors:** Roger Adam Close, Bouwe Rutger Reijenga

**Affiliations:** ^a^Department of Earth Sciences, University of Oxford, Oxford OX1 3AN, United Kingdom

**Keywords:** Paleobiology, species–area relationship, Cretaceous–Paleogene, mass extinction, spatially explicit neutral models

## Abstract

Mass extinctions are rare but catastrophic events that profoundly disrupt biodiversity. Widely accepted consequences of mass extinctions, such as biodiversity loss and the appearance of temporary “disaster taxa,” imply that species–area relationships (SARs, or how biodiversity scales with area) should change dramatically across these events: Specifically, both the slope (the rate of accumulation of new species with increasing area) and intercept (the density of species at local scales) of the power–law relationship should decrease. However, these hypotheses have not been tested, and the contribution of variation in the SAR to diversity dynamics in deep time has been neglected. We use fossil data to quantify nested SARs in North American terrestrial tetrapods through the Cretaceous–Paleogene (K/Pg) mass extinction (Campanian–Ypresian). We show that SARs vary substantially through time and among groups. In the pre-extinction interval (Maastrichtian), unusually shallow SAR slopes (indicating low beta diversity or provinciality) drive low total regional diversity in dinosaurs, mammals, and other tetrapods. In the immediate postextinction interval (Danian), the explosive diversification of mammals drove high regional diversity via a large increase in SAR slope (indicating higher beta diversity or provinciality), and only a limited increase in SAR intercept. This contradicts the expectation that postextinction biotas should be regionally homogenized by the spread of disaster taxa and impoverished by diversity loss. This early postextinction increase in SAR slope was followed in the Thanetian–Selandian (∼4.4. myr later) by increases in the intercept, indicating that diversity dynamics at local and regional scales did not change in synchrony.

## Significance

The species–area relationship (SAR), which describes how diversity scales with geographic area, is one of the few nearly ubiquitous phenomena in macroecology. In continental settings, nested SARs encapsulate both the number of species that live in a single location, and how rapidly different species’ identities turn over across space. However, despite their universality, it is unclear how they have varied in the wake of mass extinction events. Here, we show how SARs for North American terrestrial vertebrates varied across the Cretaceous–Paleogene mass extinction. After the extinction of nonavian dinosaurs, mammals initially show increases in spatial turnover followed by local increases millions of years later. This illustrates the asynchronous change in SARs and recovery dynamics of biodiversity through deep time.

The Cretaceous–Paleogene mass extinction (K/Pg) was one of the most influential events in the history of Phanerozoic biodiversity. On land, this event caused the demise of several major groups, including all nonavian dinosaurs (1–6). Leading hypotheses of the causes of the K/Pg mass extinction include a large bolide impact in the Yucatan peninsula, which resulted in abrupt large-scale environmental deterioration (2, 3), and the eruption of >1.1 million km^3^ of continental flood basalts over a period of ∼750,000 y in the Deccan volcanic province (4, 7). Counterintuitively, and perhaps uniquely among mass extinction events, the K/Pg event catalyzed rapid net increases in biodiversity, over timescales of hundreds of thousands to millions of years. The explosive radiation of mammals (8–12), in particular, resulted in a stepwise increase in the diversity of terrestrial tetrapods, establishing a new diversity equilibrium on land that was maintained throughout the Cenozoic (8, 13–16). However, we do not yet understand whether this postextinction diversity rebound was driven solely by changes in local richness, or by changes to the spatial scaling of diversity.

Past studies of terrestrial tetrapod diversity across the K/Pg have investigated patterns at a variety of discrete spatial scales, including at local (16), regional or continental (13–15), and global (17, 18) levels. However, diversity increases with the area over which it is measured (e.g., ref. 19). This ubiquitous pattern is formalized as the nested species–area relationship [SAR; (20)] which, over intermediate spatial scales, is most commonly characterized using a power-law model (19–21) of the form $S=cA^{z}$, where S is species richness, A is area, z is the slope in log–log space (the rate at which species richness increases with increasing area), and c is the species richness at one unit area. When expressed using log-transformed units as log(S)=log(c)+zlog(A), this yields a straight-line relationship where log(c) is the intercept. The form of the nested SAR reveals rich information about the spatial structure of diversity in continental settings, and especially about the links between local- and regional-scale diversity.

Variation in regional-scale (gamma) diversity is an emergent phenomenon, driven by some combination of 1) changes in richness at small spatial scales (e.g., the richness of local communities, or alpha diversity), which causes the intercept of the nested SAR to vary; and 2) changes to the rate at which diversity scales with area, which causes the slope of the nested SAR to vary. The rate at which diversity scales with area in continental settings is a function of the level of spatial homogeneity of species distributions—in fact, nested SARs follow a power-law function at intermediate spatial scales because individuals within species are spatially aggregated, rather than randomly distributed (20). The geographic turnover of species’ identities is often termed “beta diversity” or, at larger scales, provinciality (22). The slope of nested SARs is an effective way to summarize information about species’ distributions across spatial scales (20). Understanding how diversity scales with area is especially important in the fossil record, because variation in the geographic distribution of sampled fossil localities through time (“spatial bias”) is known to be substantial (14–16, 23). For this reason, it is important to explicitly quantify spatial structure when estimating biodiversity dynamics through deep time. SARs inherently control for such spatial bias, and offer insights that individual discrete spatial scales do not.

Mass extinctions profoundly impact biodiversity at all spatial scales (24–27). By disrupting and subsequently restructuring ecological communities (24–28), and by upending existing patterns of species distributions (29, 30), these events have the potential to substantially alter key macroecological phenomena, including species–area relationships. For example, the selective extinction of species with smaller geographic ranges (27, 31) would be expected to result in flatter SAR slopes, by eliminating those species that make the greatest contribution to beta diversity. Furthermore, postextinction faunas may be dominated by superabundant and highly cosmopolitan “bloom” or “disaster” taxa (28, 32, 33). If so this would be expected to result in greater spatial homogeneity of species distributions in postextinction assemblages. Both of these effects should result in flatter SAR slopes, while the loss of species at local scales might be expected to lower SAR intercepts.

A bolide impact represents the most likely trigger for the K/Pg extinction, initiating a wide range of potential kill mechanisms including wildfires, acid rain, and global cooling (1). However, longer-term environmental stressors may also have contributed to ecological disruption in the lead-up to this event (1, 34), including flood volcanism (4, 7), climate change (1, 35–38), and sea-level change (39–41). Consistent with this scenario, ecosystem modeling has suggested that Campanian–Maastrichtian dinosaur communities in North America were more vulnerable to disruption of primary productivity (34), and latest Cretaceous (Maastrichtian) dinosaur assemblages in North America exhibited low beta diversity (42).

However, despite considerable interest in the effects of mass extinctions on terrestrial communities and species’ distributions (28–30, 33, 43), and in the K/Pg in particular (e.g., refs. 11, 44, and 45), no study has directly quantified the effects of mass extinction events on terrestrial species–area relationships. In fact, few studies have quantified how species–area relationships of any kind have varied through deep time (but see refs. 46–48 for Miocene-Recent mammals). To better understand the effect of mass extinctions on macroecological patterns and processes, we need a unified understanding of how biodiversity varies across a range of spatial scales.

**Fig. 1.**
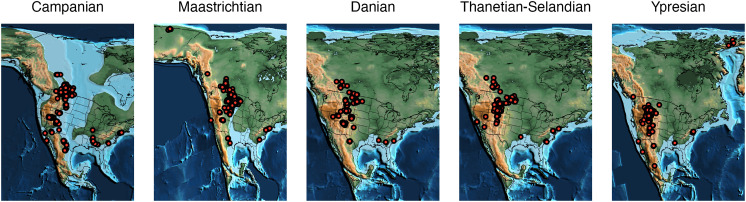
Variation in fossil sampling through time from the Campanian to the Ypresian. Red points represent coordinates of the mid-points of equal-area grid cells where terrestrial tetrapod fossils have been recovered. Paleogeographic maps were obtained from the PALEOMAP PaleoAtlas (49, 50) and cropped to the sampling extent of the fossil record.

Here, we use fossil occurrence data to analyze how species–area relationships for North American terrestrial (=nonflying, nonmarine) tetrapods, and major subgroups, varied across a window of ∼36 My around the K/Pg boundary (Campanian–Ypresian; Fig. 1). This interval encompassed the lead-up to the K/Pg extinction, its aftermath, and recovery. It witnessed substantial environmental change between the Campanian and Maastrichtian (1), including orogenic activity and sea-level change affecting the paleogeography of the Western Interior seaway (a large inland sea dividing North America into western and eastern landmasses, which existed in some form from the early Late Cretaceous until the earliest Paleocene), Deccan flood-volcanism (4), and long-term global cooling toward the end of the Cretaceous (1, 35–38); a period of dramatic climate fluctuations during the early Paleogene (51); and protracted ecosystem recovery (44) that was only complete by the early Eocene (52). We focus on the North American tetrapod record from this interval because it is intensively studied, well-documented, and has consistently broad geographic scope. We reconstruct species–area relationships using progressively nested sets of adjacent spatial points (locations containing fossil localities), with spatial extent for each subregion quantified as summed minimum-spanning tree (MST) length (the shortest possible set of connections between all spatial points; see *Materials and Methods*). We estimate diversity use Shareholder Quorum Subsampling [SQS (53, 54); see *Materials and Methods*], which draws down all diversity samples to the same prespecified level of sample completeness, or “coverage” of the species-abundance distribution (*Materials and Methods*). By comparing empirical SARs with geographically scrambled null distributions and Spatially Explicit Neutral Models (SENMs; see *Materials and Methods*), we show that SARs reconstructed using SQS diversity estimates are reliable, while those using unstandardized species counts are not (*Materials and Methods*). By quantifying variation in the form of the SAR, we are able to determine which spatial scales drove overall patterns of biodiversity change over this key interval, and variation among major taxonomic groups, including nonavian dinosaurs, mammals, and other taxa.

## 1. Results

**Fig. 2.**
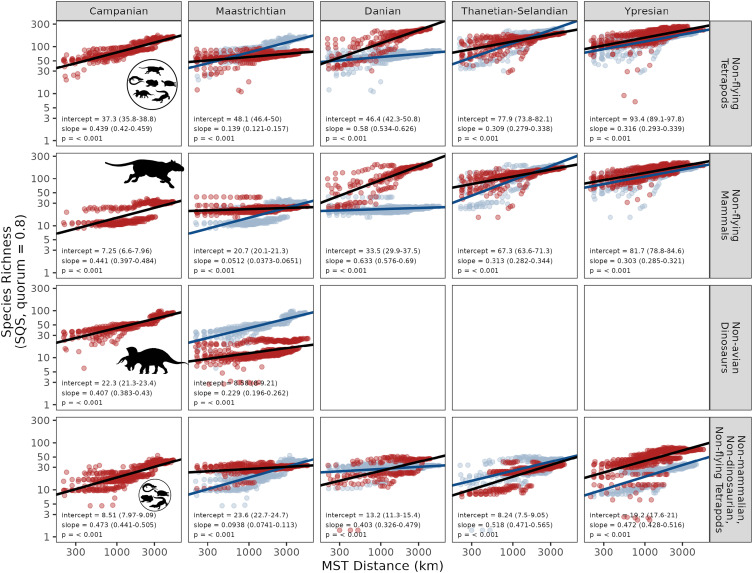
Species–area relationships for North American terrestrial tetrapods and major subgroups across the Late Cretaceous–early Paleogene. Columns represent the composite time bins, and rows represent the taxonomic groups. Each facet shows the nested species–area relationship for the respective group and bin (red), and the previous bin if relevant (blue). Each data point represents an unique nested set of contiguous (adjacent, or nearest-neighbor) fossil localities, and area is measured as the minimum distance (km) that connects all localities (the summed minimum spanning tree length, or MST; see *Materials and Methods*). Species richness estimates for each nested set were obtained using SQS at a quorum of 0.8. Ordinary least-squares regression fits and estimated parameters are shown for the current (black) and previous bin (blue).

**Fig. 3.**
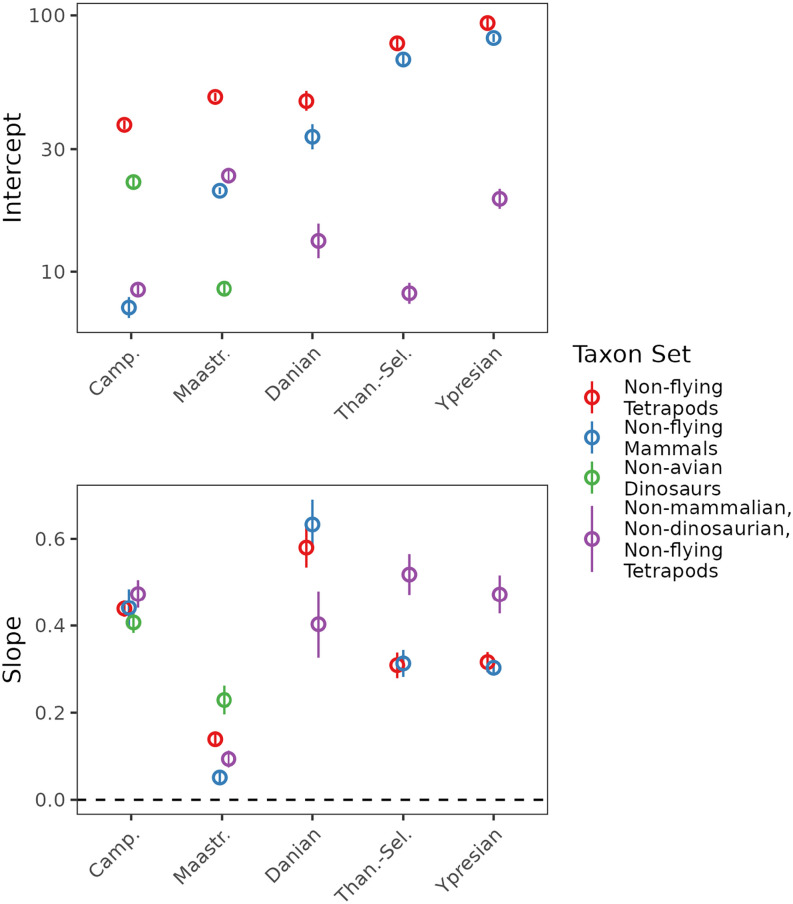
Slope and intercept parameters for species–area relationships (richness estimates obtained using SQS at a quorum of 0.8) for North American terrestrial tetrapods and major subgroups across the Late Cretaceous–early Paleogene.

Our results show a substantial shift in the SAR for terrestrial tetrapods over the K/Pg boundary. The slope of the SAR increased abruptly during the earliest Cenozoic (Danian; Figs. 2 and 3). This indicates that species were much less homogeneously distributed in the Danian than they were during the latest Cretaceous (Maastrichtian), being instead more similar to that seen in the preceding Campanian interval. Tetrapods as a whole show little change in intercept into the Danian, but in mammals the increase in slope is accompanied by a near-twofold increase in intercept (2 and 3) (consistent with ref. 16). These pronounced increases in slope and intercept for mammals during their postextinction radiation jointly explain the large increase in regional-scale diversity for terrestrial tetrapods as a whole in the earliest Cenozoic. Mammals ultimately experienced a fourfold rise in the intercept of their SAR between the Maastrichtian and the Ypresian (Figs. 2 and 3), indicating a quadrupling of their diversity within local communities into the Eocene. Patterns in “minor” groups (clades with lower diversity or less well-sampled fossil records) are less clear-cut (*SI Appendix*, Fig. S1), either because they are characteristically species-poor (crocodilians, turtles) or because their fossil records are sparser, even if true diversity was likely high (squamates, lissamphibians). Because of their data limitations, we focus primarily on the interpretation of the major groups and aggregated groups. However, it is clear that none of these other groups experienced the rapid and emphatic postextinction rebound seen in mammals.

We also find evidence for a comparatively short-lived departure toward a substantially shallower slope during the latest Cretaceous (Maastrichtian), indicating a substantial decrease in beta diversity or provinciality before the K/Pg boundary (Figs. 2 and 3 and *SI Appendix*, Fig. S1; statistical tests of significance in slope between successive pairs of time bins are given in Table 1). Slopes for SARs of terrestrial tetrapods as a whole (Figs. 2 and 3) decreased from ∼0.439 in the Campanian to 0.139 in the Maastrichtian, and 95% CIs do not overlap. SAR slopes were also shallower during the Maastrichtian in nearly every major tetrapod group, including dinosaurs, lissamphibians, mammals, squamates, and turtles, and when aggregating minor groups (“nonmammalian, nondinosaurian, nonflying tetrapods;” *SI Appendix*, Fig. S1). The unusually shallow SAR slope in the Maastrichtian produces the lowest regional-scale diversity of all the intervals examined here—for terrestrial tetrapods, dinosaurs, squamates, and to a lesser degree, mammals. Intercepts of SARs show more variation among groups. In tetrapods as a whole, the intercept increases slightly from the Campanian to the Maastrichtian, although diversity estimates for individual subsampled spatial regions broadly overlap between the two intervals. Dinosaurs and mammals, however, show opposing patterns, with a modest increase in intercept for mammals and a large decrease in dinosaurs.

Although the SAR slope did increase into the Danian following the K/Pg, longer-term increases in regional species richness between the Mesozoic and Cenozoic result more strongly from increases in intercept rather than slope. While large and statistically significant differences are recovered between slopes for the Maastrichtian and Danian, slopes are very similar between the Campanian and either the Thanetian–Selandian or Ypresian. Furthermore, Early Paleocene (Danian) increases in the slopes of the SARs for tetrapods and mammals were followed by an increase in the intercept and a sharp relaxation of the slope to lower levels later in the Paleocene (Thanetian–Selandian). Subsequent changes in slope and intercept for tetrapods and mammals between the Thanetian–Selandian and Ypresian are relatively minor, despite dramatic fluctuations in climate [e.g., the Paleocene–Eocene Thermal Maximum (51)]. While the slope decreases significantly from the Danian to the Thanetian–Selandian, from the Thanetian–Selandian to the Ypresian, the slope in tetrapods and mammals remains stable. In contrast, intercepts increase modestly, resulting in slightly higher gamma diversity at the largest comparable spatial scales.

Although our SENM simulations show that we can reliably estimate SAR slopes and intercepts from empirical distributions of fossil localities when all samples are contemporaneous (*SI Appendix*, Figs. S2–S5; see *Materials and Methods*), the temporal resolution of the fossil data in our focal analyses might nevertheless influence our findings. Specifically, temporal turnover of faunas sampled within geological stages might cause varying degrees of temporal “pileup” of diversity (lumping together species that did not coexist), and when reconstructing SARs, these effects could interact with temporal changes in the spatial distribution of fossils within bins. For example, the relatively short duration of the Maastrichtian (6.1 Ma, vs. 11.5 Ma for the Campanian) might explain the shallow slope if this translated into less temporal turnover of species’ identities. Conversely, rapid temporal turnover in the wake of the K/Pg might explain an increased slope in the Danian, although the magnitude of taxonomic turnover within bins would not be tightly linked to temporal duration if it were pulsed at bin boundaries rather than continuous [e.g. as shown for marine taxa (55)].

To investigate the effects of temporal resolution, we also binned the fossil occurrences at the level of substage in the Cretaceous and North American Land Mammal Ages [NALMAs, chronostratigraphic units defined based on cohorts of coexisting species that do not map neatly onto geological stages (56)] in the Paleogene (*Materials and Methods*). Binning at this more finely resolved temporal resolution comes at the cost of data quantity and spatial coverage within bins, and SENM simulations show that certain intervals (Early Campanian, Early Maastrichtian, Clarkforkian, Uintan) lack sufficient data to adequately reconstruct species–area relationships (*SI Appendix*, Figs. S6–S9). However, the remaining intervals reveal similar overall trends in SAR slope and intercept to those obtained using geological stages (*SI Appendix*, Figs. S10–S12 and Table S1), including a shallower slope in the Middle–Late Maastrichtian substage, a steeper slope, and increased intercept immediately following the K/Pg (Puercan and Torrejonian), followed a gradual transition to a shallower slope and higher intercept by the end of our study interval (Bridgerian).

## 2. Discussion

By presenting direct reconstructions of species–area relationships across a major extinction event, we show that changes in the spatial scaling of species richness caused biodiversity dynamics at local and regional scales to become decoupled through time. Changes in spatial scaling played an important role in the dynamics of recovery from the end-Cretaceous mass extinction.

### 2.1. Decrease in Species–Area Slope in the Maastrichtian.

An especially shallow slope is observed in the latest Cretaceous (Maastrichtian), implying low beta diversity during the lead-up to the mass extinction. This contrasts with substantially steeper slopes, implying higher beta diversity, during the Campanian and earliest Cenozoic (Danian), an effect that is observed across all taxonomic groupings (Fig. 2 and *SI Appendix*, Fig. S1). In terrestrial tetrapods as a whole, the shallower slope primarily reflects lower regional-scale diversity relative to the Campanian (i.e., little change in diversity at small spatial scales, but less spatial turnover in species’ identities; Fig. 2), while in mammals and other nonmammalian, nondinosaurian tetrapods, it reflects only a modest decrease in regional-scale richness, coupled with a large increase in richness at small spatial scales (and therefore less spatial turnover in species’ identities; *SI Appendix*, Fig. S1).

Dinosaurs, meanwhile, show a pronounced decrease in both the slope and intercept of their SAR in the Maastrichtian. This is a feature that cannot easily be discerned by estimating diversity only for narrow-ranging, discrete spatial scales (*SI Appendix*, Fig. S1). However, this finding is consistent with a previously documented decrease from the Campanian to the Maastrichtian in the local (16) and regional-scale (15) richness of North American dinosaurs. Vavrek and Larson (42) also suggested that beta diversity in Maastrichtian dinosaurs of western North America was low. However, this inference was made by comparing Maastrichtian beta diversity to extant North American birds, and without reference to other fossil data. Here, by contrast, we show that beta diversity (as measured by the SAR slope) in dinosaurs decreased from the Campanian into the Maastrichtian, and that other tetrapod groups experienced similar decreases over this interval. To provide broader context for these results, future work could compare our findings against SARs for dinosaurs in other geographic regions and time intervals, although sparse data (especially adequate spatial coverage) may limit robust estimates.

This shift toward a shallower slope in the Maastrichtian may capture a true biological signal of decreasing beta diversity or provinciality, perhaps driven by tectonic (37) or climatic changes (4) from the preceding Campanian. The spread of angiosperm-dominated ecosystems during the Cretaceous Terrestrial Revolution [KTR; 125 to 80 Ma (57, 58)] could conceivably have resulted in the creation of novel niches, as well as the disappearance of old niches associated with gymnosperms. If such niche changes primarily affected alpha diversity, this would be consistent with the observed increase in the intercept for mammals and decrease for dinosaurs between the Campanian and the Maastrichtian. However, the decrease in slope observed in both groups, and the stability of regional diversity in mammals, suggests an alternative explanation. The Sevier and Laramide orogenies, in conjunction with sea-level change, likely formed an allopatric barrier that separated western and eastern faunas in the Campanian, but which was subsequently removed by a marine transgression in the Maastrichtian (37). Homogenization of previously separated faunas could lead to lower beta diversity, and thus shallower species–area slopes. Deccan flood volcanism (4) and consequent environmental changes might also play a role, although the specific effects that this event might have had on Maastrichtian spatial biodiversity patterns are unclear (1). However, it is also possible that these trends may reflect differences in the spatial, environmental, and temporal structure of the sampled Campanian and Maastrichtian fossil records of North America (59, 60), rather than true shifts in the spatial structure of biodiversity. By explicitly quantifying nested species–area relationships, we have addressed issues arising from differences in spatial coverage between stages. We have also investigated some effects of temporal structure using more finely resolved time bins (*Results*), so this is unlikely to be the explanation. Future analyses could combine our approach with more detailed information on environmental and depositional settings, and taphonomic conditions, to investigate these possibilities.

**Table 1.** OLS slope changes between time bins

Contrast	Null.value	Estimate	SE	df	Statistic	*P* value	Significance
Nonflying tetrapods							
Campanian - Maastrichtian	0	0.300	0.017	2,684	17.858	0.000	<0.01
Maastrichtian - Danian	0	−0.441	0.021	2,684	−21.133	0.000	<0.01
Danian - Thanetian–Selandian	0	0.271	0.022	2,684	12.105	0.000	<0.01
Thanetian–Selandian - Ypresian	0	−0.008	0.018	2,684	−0.428	0.993	n.s.
Nonflying mammals							
Campanian - Maastrichtian	0	0.390	0.021	2,563	18.540	0.000	<0.01
Maastrichtian - Danian	0	−0.582	0.023	2,563	−25.378	0.000	<0.01
Danian - Thanetian–Selandian	0	0.320	0.024	2,563	13.374	0.000	<0.01
Thanetian–Selandian - Ypresian	0	0.010	0.019	2,563	0.529	0.984	n.s.
Nonavian dinosaurs							
Campanian - Maastrichtian	0	0.178	0.023	1,031	7.776	0.000	<0.01

### 2.2. Patterns Across the K/Pg Boundary.

We show that the end-Cretaceous mass extinction catalyzed increases in both the intercept and slope of the SAR in North American terrestrial tetrapods at the temporal resolution of our study (Fig. 2). The SAR slope underwent a large, early increase between the Maastrichtian and the Danian, followed by a subsequent increase in the SAR intercept, and relaxation of the slope, up to 4.4 My later (between the Danian and Thanetian–Selandian; Fig. 2). This temporal decoupling of SAR slope and intercept suggests a substantial contribution of variation in beta diversity in shaping the dynamics of regional diversity change in North America across the K/Pg mass extinction and recovery interval.

The early and transient increase in slope indicates high within-region provinciality in the aftermath of the K/Pg. This differs from expectations based on prior knowledge of mass extinction events in general, including biodiversity loss [especially if it results from the selective extinction of species with smaller ranges (11, 27, 31)] and the occurrence of “disaster taxa” (10, 33, 45, 61), which would be expected to flatten SAR slopes and decrease intercepts. In contrast, the K/Pg seems to be characterized by lower provinciality before the event and a large increase in intercept, and our results are therefore inverted from theoretical expectations. However, the recovery dynamics we document took place over longer timescales than those investigated by some previous studies, which document changes in diversity and faunal composition over tens to hundreds of thousands of years, albeit for a much more restricted spatial scope (10, 44, 45, 61).

If sufficient fossil data were available to reconstruct species–area relationships over these high-resolution timescales, it is possible that we would observe different recovery dynamics in the immediate aftermath of the extinction. For instance, species–area relationships might have been flatter prior in the very earliest phases of recovery, following the range-expansion of survivors but prior to the origination of novel species. Nevertheless, recovery from the end-Cretaceous was much more rapid than other mass extinctions (11), such as the end-Permian (25, 28). While disaster taxa have been documented during the first 100 to 200 k.y. following the end-Cretaceous (10, 61), recovery from the end-Permian was much slower, with disaster taxa like *Lystrosaurus* dominating for millions of years (33), perhaps due to the persistence of unfavorable environmental conditions (25, 28).

The increase in intercept and slope between the Maastrichtian and the Danian reflects modestly higher tetrapod diversity at small (∼100 km MST distance) spatial scales, coupled with more substantial increases at large (>1,000 km MST distance) spatial scales. These changes are driven by a pronounced increase in the mammalian SAR intercept. Other tetrapod groups examined here did not experience a rapid postextinction rebound (Fig. 2 and *SI Appendix*, Fig. S1), consistent with previous findings for groups such as squamates (62) and nonmarine crocodylians (63). Although we do not analyze patterns for flying tetrapods (i.e., pterosaurs, birds, and bats) because their fossil records are inadequate for most regions and intervals, it is conceivable that flying taxa could have impacted the dynamics of SARs for tetrapods as a whole, especially given the explosive radiation of birds in the early Cenozoic (64).

Our findings are consistent with recent work on tetrapod species richness at local to regional spatial scales, which documented twofold to fourfold increases in terrestrial tetrapod diversity in the earliest Cenozoic, driven by the explosive radiation of mammals (10, 13–16, 44, 61, 65). Together, this work suggests that diversity equilibria for terrestrial tetrapods were reset at local and/or regional spatial scales, as well as an important role for changes in the spatial scaling of biodiversity over the postrecovery interval. A similar phase-shift toward higher diversity across the K/Pg was recently documented for marine animals at regional spatial scales, driven by the explosive radiation of gastropods (23). Taken together, these results contribute to a revised understanding of the macroevolutionary role of the end-Cretaceous extinction. The emerging picture contradicts classical expectations that mass extinctions primarily eliminate biodiversity, and instead suggests that the K/Pg mass extinction was a major generative force, ultimately creating higher levels of biodiversity—in large part due to exceptional radiations by a small number of taxonomic groups—that persisted through the Cenozoic.

## 3. Materials and Methods

### 3.1. Data.

Fossil occurrence data for terrestrial tetrapods (i.e., nonflying, nonmarine groups) were downloaded from the Paleobiology Database (http://www.paleobiodb.org/) on 17 November 2023. All procedures for downloading and processing occurrence data (e.g., to exclude unsuitable records) are identical to those described in ref. 15, except that the analysis was restricted to North America over the interval spanning the Campanian to the Ypresian. As part of this procedure, the occurrence records were parsed into sets for major tetrapod subgroups (“dinosaurs” = Dinosauria excluding Aves; “mammals” = Mammaliamorpha excluding Chiroptera; “squamates” = Squamata, “crocodilians” = Crocodylomorpha; “turtles” = Testudinata; “lissamphibians” = Lissamphibia). Flying taxa (Aves, Chiroptera, and Pterosauria) were excluded because their fossil record is inadequate for most intervals and regions, and dominated by Lagerstätten deposits (14–16). We take the lack of information about flying taxa into account when interpreting our results. The analysis was conducted at species-level. The final cleaned occurrence dataset comprised 14,887 occurrences from 3,766 collections, representing 1,661 species. Occurrence data were binned into nominally equal-length composite time bins, created by combining stratigraphic stages, following ref. 15. Over our study interval, however, only the Thanetian–Selandian bin comprises more than a single stratigraphic stage (*SI Appendix*, Table S2). To test the effects of the temporal resolution of our binning scheme, we also conducted the analysis using a combination of geological substages [NALMAs; (56, 66)]. Although NALMAs are recognized for the Cretaceous, the occurrence data in the PaleoDB are only resolved to substage level. Occurrence data spanning the interval from the Early Campanian to the Uintan (a NALMA interval contained within the Lutetian stage) were binned using the early_interval and late_interval fields of the PaleoDB download (*SI Appendix*, Table S3).

### 3.2. Spatial Subsampling.

We reconstructed nested SARs by estimating diversity within spatial regions of known paleogeographic extent, as calculated from the paleocoordinates of fossil localities. Each subsampled spatial region consists of a set of geographically adjacent fossil localities. Following our previous work on spatially explicit diversity estimation (14, 15, 23), we used MST distance in kilometers as our measure of spatial extent (see ref. 14). Subsampled spatial regions were constructed using an algorithm (modified from ref. 67) that identifies all unique nested sets of directly adjacent fossil localities. To reduce computational complexity, paleocoordinates for fossil localities were binned into 100 km equal-size hexagonal/pentagonal grid cells [using the R package dggridR (68)] prior to spatial subsampling. Midpoints of these 100 km grid cells were used to define the spatial points used in our spatial subsampling algorithm. Our algorithm comprises the following steps: 1) first, a spatial point is randomly chosen as a starting location; 2) the nearest spatial point is then identified (chosen at random if there are two or more equidistant points), and these two points are used to define the first subsampled spatial region; 3) the nearest spatial point to the first two points is identified, and this set of three points is used to define the second subsampled spatial region; and 4) this procedure continues, point by point, until all spatial points have been added. This procedure is then repeated using every possible starting location, and any duplicate paleogeographic regions are removed. Our implementation of this algorithm is identical to that described in refs. 15 and 23, and full details are given there. Note that we did not use the subsequent region-clustering steps employed by Close et al. (15, 23), as these are only suitable for analyzing regional-scale diversity at discrete spatial scales. To avoid estimating diversity from regions containing widely separated clusters of localities, we screened our regions to ensure that the maximum nearest-neighbor distances between localities was less than 1,000 (*SI Appendix*, *Supplementary Methods*). Using this spatial subsampling procedure, we identified a total of 2,792 distinct regions between ∼150 km and ∼5,500 km MST distance across all time intervals.

### 3.3. Identifying Contiguous Regions for Estimating Species–Area Relationships.

Quantifying SARs for sets of fossil localities which span multiple distinct spatial aggregations (i.e., in which groupings of localities are separated by longer distances devoid of sampling) is undesirable, because multiple distinct relationships are more likely to be superimposed. To avoid this, we identified distinct spatial aggregations of fossil localities using MSTs. For each interval, a global MST was calculated using the paleocoordinates of the fossil localities (in this case, the “global” MST only encompasses North America; Fig. 1). The global MST was then split into subtrees by removing branches longer than 1,000 km. Subtrees with fewer than 10 MST nodes (i.e., binned paleocoordinates from which fossil localities are known) were excluded from the analysis due to inadequate data. In our case (North America from the Campanian to the Ypresian), this resulted in just one SAR per equal-length bin. Only subsampled spatial regions that were entirely contained within the identified subtrees were used to construct SARs.

### 3.4. Diversity Estimation.

To control for heterogeneous sampling intensity when estimating diversity for each subsampled spatial region, we use Shareholder Quorum Subsampling [SQS (53, 54, 69–71)], implemented using the R package iNEXT (72). SQS draws down all diversity samples to the same prespecified level of sample completeness, or “coverage” of the species-abundance distribution [sample coverage ranges between 0, meaning no coverage, and 1, meaning complete coverage; the target level for standardization is called the “quorum” value (73)]. Standardizing to equal coverage tells us how many species would be found on average in a random sample of a prespecified fraction of individuals (determined by the chosen quorum value) drawn from the underlying population. We focus on results using a quorum of 0.8 (i.e., the number of species observed on average in a random sample of 80% of the individuals in the underlying population), which achieves a good balance between retention of data points and richness estimator performance. For comparative purposes, face-value counts of species (=“raw” or uncorrected species richness) were also calculated for each subsampled spatial region. However, by comparing empirical SARs with geographically scrambled null distributions (see below) and Spatially Explicit Neutral Models (SENMs; see below), we show that SARs reconstructed using face-value (=“raw” or “unstandardized”) species counts are misleading: Due to undersampling, they predominantly represent sample-accumulation curves, and cannot therefore be interpreted as real biological species–area relationships. In contrast, SARs estimated using SQS do not suffer from this problem (*SI Appendix*, Fig. S13), and so we focus on results using SQS.

### 3.5. Estimating Empirical Species–Area Relationships.

SARs were constructed by plotting diversity estimates for each subsampled spatial region as a function of paleogeographic extent (km MST distance) with log–log axes. The form of the SAR was characterized using ordinary least-squares (OLS) regression, as per (67). The slope of the relationship corresponds to the *z* parameter in the classic power-law equation describing linear SARs, and the intercept to the *c* parameter (19, 74).

The slope of the power–law relationship in a nested SAR can be straightforwardly interpreted as the average rate of scaling of diversity with area and of average beta diversity across the spatial scales being analyzed. However, the intercept of the nested SAR cannot be solely interpreted in terms of changes in local richness. There are two reasons for this. First, in this study, the smallest spatial scales quantified (∼100 km summed MST distance) are larger than those needed to quantify local richness. As a result, beta diversity at very small scales (e.g., among adjacent habitats) could drive changes in the intercept even if local richness *perse* did not change. Second, changes to the slope can also affect the estimated intercept at zero kilometers, even if richness at the smallest measured spatial scales did not change. For example, increasing the slope will also decrease the intercept at zero kilometers, while diminishing the slope will increase the intercept, even in the absence of any change in the species richness observed for the smallest subsampled spatial regions. For these reasons, we do not use the traditional estimate of the intercept (i.e., the slope of the regression line at zero), and instead use the estimate at the smallest measured spatial scale of 100 km. For convenience, we refer to this as the “intercept,” and use it as an estimate of local diversity at a scale of approximately 100 km. In addition, very small spatial scales lie outside the Arrhenius Zone [the range of spatial scales in nested SARs where linear changes in diversity as a function of area are observed in log–log space (19, 20, 74)], and at smaller scales, a nonlinear relationship is observed (20). For this reason, the intercept of the SAR should here be interpreted as diversity at the smallest measured spatial scales, rather than local richness in the strict sense.

We tested for differences in slopes of successive bins using an interaction with covariates analysis implemented via the function “emtrends()” in the R package “emmeans.”

### 3.6. Null Distributions.

Incomplete sampling of diversity introduces a signal of sample-accumulation to nested SARs, which is independent of the true relationship between diversity and area. This results from increasing sample coverage at larger spatial scales and may exaggerate slopes estimated from empirical data, especially when using face-value counts of species (=raw or uncorrected species richness). To assess the potential impacts of this, we compared our empirical SARs to a null distribution generated by randomizing the spatial structure of the fossil record. This was achieved by randomly reassigning the paleocoordinates of collections within each bin, without replacement, to remove the effects of spatial structure and beta diversity. This allowed comparison of our empirical regression relationships to those based on null distributions in which no SAR should exist, except due to the effects of sample incompleteness.

These null distributions show that SARs estimated using face-value taxon counts contain a strong sample-accumulation curve component (*SI Appendix*, Fig. S13). Using face-value richness, the median slope of the null distribution is almost always comparable to the empirical slope, even though no relationship between richness and area should exist. In contrast, sampling-standardized SARs estimated using SQS do not suffer from this problem (*SI Appendix*, Fig. S13). Null distributions using SQS are, on average, almost perfectly flat, correctly inferring that no relationship between diversity and area exists. Conversely, the empirical relationships estimated using SQS are much steeper than their corresponding null distributions. We therefore focus our interpretation entirely on patterns estimated using SQS.

Furthermore, null distributions for minor groups, even when aggregated (nonmammalian, nondinosaurian, nonflying tetrapods), suggest that SAR parameter estimates for some intervals may be less reliable than those for the major groups (e.g. Thanetian–Selandian, *SI Appendix*, Fig. S13). For this reason, we focus our interpretation more on the major groups (nonflying tetrapods, mammals, and dinosaurs).

### 3.7. Spatially Explicit Neutral Models.

The fossil record shows variation through time in the number of localities sampled, their geographic dispersion, and the intensity with which they are sampled. Our ability to infer changes in SARs through geological time may be impacted if sampling is not sufficiently extensive or complete. We used spatially explicit neutral models [SENMs; (75, 76)] to assess whether the spatial sampling structure of the empirical fossil record is sufficient to infer the shape of and changes in SARs through time. SENMs allow for the efficient generation of assemblages of many individuals, from small islands to continents, and have been shown to generate realistic SARs (75, 77, 78). Assemblages simulated across the entirety of the North American subcontinent could then be subsampled to reflect the sampling extent and intensity of the fossil record, such that we know both the true and subsampled SAR. Our goals were to investigate whether there was 1) any inherent bias in estimation of SAR slope and intercept caused by variation in fossil-record sampling through time, 2) if local-to-global SARs could be accurately inferred from regional fossil SARs, 3) if excluding clusters of localities with a nearest-neighbor distance greater than 1,000 km was justified, and 4) to further investigate whether face-value counts of species or those estimated using SQS were more accurate.

We briefly summarize the results of these analyses here (full details are provided in *SI Appendix*). 1) We found that face-value counts lead to misleading estimates of both slopes and intercepts (consistent with the results of our null distributions, above), showing sudden increases or decreases when the actual slopes and intercepts show no trend (*SI Appendix*, Figs. S2–S5). 2) We also found that if slopes and intercepts are estimated from only the geographic cells that contain fossils (rather than using the full output of the simulation across the entire continental area), the estimates do not show an apparent bias (*SI Appendix*, Figs. S2–S5). 3) When we downsample these cells to reflect the sampling heterogeneity through space (e.g. variation in the number of collections and occurrences), and correct for this unevenness with SQS, we find that both slope and intercept are underestimated systematically. However, no apparent bias arises in the estimates. The Danian shows greater variability than any of the other time bins. The estimates become more precise when dispersal distance increases (*SI Appendix*, Figs. S2–S5). 4) Including long branches (i.e., grouping clusters of cells located further than 1,000 km from any other cells) causes estimations to poorly reflect the slopes and intercepts of SARs against which they are compared (as they cover a larger area for which there is limited information; *SI Appendix*, Figs. S2–S5). 5) Last, a comparison between local-to-continental SARs and the regional SARs estimated from the fossil sites (completely sampled), shows that although the regional SAR shifts with changing parameter values (reflecting the continental trends), the regional SAR has a limited ability to pick up all continental trends, often overestimating the intercept and underestimating the slope, as more species-poor areas are not captured. (*SI Appendix*, Figs. S14–S17).

## Supplementary Material

Appendix 01 (PDF)

## Data Availability

Fossil occurrence data from Paleobiology Database and full analysis code have been deposited in FigShare (DOI: 10.25446/oxford.28303568) (79).
